# Corpus callosum microstructure in chimpanzees (pan troglodytes): associations with age, handedness and cognition

**DOI:** 10.3389/fnagi.2025.1611611

**Published:** 2025-09-03

**Authors:** Maëlig Chauvel, Ivy Uszynski, Cyril Poupon, William D. Hopkins

**Affiliations:** ^1^BAOBAB, NeuroSpin, Université Paris-Saclay, CNRS, CEA, Gif-Sur-Yvette, France; ^2^Department of Comparative Medicine, Michale E. Keeling Center for Comparative Medicine and Research, The University of Texas MD Anderson Cancer Center, Bastrop, TX, United States

**Keywords:** primates, corpus callosum, brain aging, diffusion MRI, chimpanzee white matter

## Abstract

Studies on the human brain have emphasized the loss of gray matter volume and decreased thickness during normal aging, along with variations in the density of small axon fibers across different regions of the corpus callosum (CC). Here, we investigated age-related changes in white matter connectivity in the CC and their association with handedness and cognitive decline in chimpanzees. To this end, microstructural measures of CC morphology were obtained from a sample of 49 chimpanzees. Initial assessments included quantifying streamline density, fractional anisotropy (FA), axial diffusivity (AD), and radial diffusivity (RD) values, which were then correlated with age and cognitive measures using the Primate Cognition Test Battery. We found an inverse association between streamline density and age in chimpanzees, particularly in the anterior and central CC regions. We also found an inverse association between FA and age in the splenium. Lastly, after controlling for age and sex, chimpanzees with higher cognition values also had higher FA values in anterior regions of the CC. Collectively, our results show that chimpanzees diverged from the typical human pattern, suggesting stronger interhemispheric connectivity integrity in frontal cortical brain regions compared to humans.

## 1 Introduction

The corpus callosum (CC) is a large set of white matter fibers that connect homo- and heterotopic regions between the left and right cerebral hemispheres. Among primates, including humans, fibers passing through the CC connect regions along an anterior-to-posterior gradient (Hofer and Frahm, 2006; Hofer et al., 2007; Pandya and Seltzer, 1986; Phillips and Kochunov, 2011; Phillips et al., 2007); thus, anterior regions of the CC correspondingly connect largely areas in the frontal lobe whereas more posterior CC regions connect cortical areas in the parietal and occipital lobes. Studies in postmortem brains have shown that the density of small and large fibers axons vary between CC regions (Pandya and Seltzer, 1986). Notably, in human and non-human primates, for the anterior and posterior regions, there is a higher density of small compared to large axon fibers whereas in the central CC regions, the ratio of small to large axon is lower (Aboitiz et al., 1992; Tomasch, 1954). Because large diameter fibers are more myelinated, this result has been interpreted as indicating that conduction times between frontal and occipital association cortex are slower than between regions involved in primary motor and sensory functions (Phillips et al., 2015). Moreover, it has also been suggested that variation in the axon diameter as a proxy to conduction delays between species may have some implications for the evolution of lateralization in structure and function in brain and behavior (Aboitiz et al., 1992; Innocenti et al., 2010; Oliveras et al., 2001; Ringo et al., 1994). However, a recent large-scale comparative study across mammals found only partial support for this hypothesis, suggesting that the relationship between brain size, conduction delays, and hemispheric asymmetries could be more complex than previously assumed (Ocklenburg et al., 2023). The significance of the CC to individual variation in lateralization for motor and cognitive functions has been eloquently demonstrated by research on split-brain patients, in which the CC is partially or completely severed there by disconnecting the two halves of the brain (Gazzaniga, 2000).

Advances in the study of CC morphology have been aided tremendously by the advent of modern *in vivo* structural and functional imaging technologies. In particular, the development of diffusion weighted imaging (dMRI) during the 1990s has provided the means to non-invasively investigate white matter microstructure. The dMRI contrast stems from the anisotropy of the displacement of water molecules within brain tissues whose trajectories are restricted or hindered by cell membranes populating them (Basser et al., 1994). A plethora of dMRI models have been proposed to model the diffusion process of water all aiming to describe the complexity of the cellular organization of brain tissues (Tournier et al., 2011). The first model, diffusion tensor imaging (DTI), was applied to characterize white matter and successfully used to infer the structural connectivity which gave rise to tractography (Le Bihan et al., 1986). Despite some limitations (for instance the inability to well present fiber crossings or the lack of specificity of its rotation invariant features such as fractional anisotropy (FA), radial diffusivity (RD) and axial diffusivity (AD)), DTI remains widely used in clinical applications due to its simple and fast acquisition scheme (Basser et al., 1994; Behrens et al., 2007; Mangin et al., 2013; Mori and Zhang, 2006; Poupon et al., 2000; Reisert et al., 2011). In addition, DTI remains particularly relevant to characterize corpus callosum at the level of the mid-sagittal plane because the axonal fibers composing it are mostly perpendicular to this plane. This constitutes a single width fiber population that respects the DTI Gaussian assumption at first approximation. For this reason, local FA, RD and AD measures can therefore be used to characterize the CC microstructure (Bennett et al., 2010).

Of specific interest to this study are reports on the association between age, fiber number, FA, AD and RD within the CC (Sullivan and Pfefferbaum, 2006). In humans, one consistent result across studies is an inverse association between age and FA values from different regions of the CC (Sullivan and Pfefferbaum, 2006). Notably, most studies report that negative correlations between age and FA are higher in the anterior regions of the CC compared to the posterior areas (Lebel et al., 2010; Matijevic and Ryan, 2021; Pietrasik et al., 2020; Sullivan et al., 2006; Teipel et al., 2014). For other microstructural measures, the results are largely similar to those reported for the FA values. For instance, Sullivan et al. (2006) reported that older human subjects had lower FA, lower fiber counts and higher RD values compared to younger individuals, and these associations were stronger in the anterior compared to posterior regions of the CC.

Investigation of white matter changes with age in non-human primates has been largely unstudied (Makris et al., 2007), particularly in chimpanzees and other great apes using DTI imaging methods (Lacreuse et al., 2005; Peters et al., 2001; Pope et al., 2020; Sherwood et al., 2011; Wisco et al., 2008). With specific reference to the CC, recent studies in baboons and chimpanzees have reported a lack of age-related loss in the surface area or thickness of any region (Hopkins et al., 2022; Westerhausen et al., 2021; Westerhausen and Meguerditchian, 2021). In a sample of 219 chimpanzee MRI scans ranging from 9 to 54 years of age, whole brain white matter volume showed a small but significant quadratic relationship with age (Autrey et al., 2014) which was not replicated in a smaller sample (*n* = 36) of female apes (Chen et al., 2013). In a sample of 36 female chimpanzees, Chen et al. (2013) reported a quadratic relationship between age and whole brain FA, RD and MD values. For FA, older and younger apes had lower values than middle-aged whereas opposite trends were found for RD and MD measures.

To further examine age-related changes in white matter, we obtained microstructural measures of CC morphology in a sample of 49 chimpanzees. With this cohort of apes, we initially quantified streamline density, FA, AD, RD and MD values and assessed their associations with age while controlling for sex and handedness of the apes. Previous studies have reported that sex and handedness are associated with corpus callosum surface area in chimpanzees (Hopkins et al., 2007; Hopkins et al., 2016; Phillips et al., 2013a; Westerhausen et al., 2021); thus, it was important to include these variables in the statistical analyses. We hypothesized that chimpanzees would exhibit a comparable pattern of selective anterior-to-posterior decline in CC integrity with age as has been reported in humans.

In addition, we also tested for associations between cognition and CC microstructure while controlling for sex, handedness and age. Notably, the chimpanzees in this study had been previously tested on the Primate Cognition Test Battery (PCTB), a 13-item test that is designed to assess physical and social dimensions of cognition in apes and monkeys (Herrmann et al., 2007; Herrmann et al., 2010a; Herrmann et al., 2010b; Hopkins et al., 2014; Russell et al., 2011; Schmitt et al., 2011). In humans, there are numerous studies reporting associations between different cognitive functions and whole brain measures of white matter microstructure as well as connectivity for specific tracts connecting different brains regions (Holleran et al., 2020; Roberts et al., 2013; Stammen et al., 2023). By contrast, there are very few studies in non-human primates including chimpanzees that have examined associations between white matter microstructure, cognition and behavior. In one study, Hecht et al. (2017) found that chimpanzees that passed the mirror self-recognition test differed in white matter volume of the superior longitudinal fasciculus as well as the gray matter volume of their terminations in the inferior formal gyrus compared to chimpanzees that failed. Latzman et al. (2015) have reported that increased volumetric connectivity between the caudate and regions within the prefrontal cortex were significantly higher in chimpanzees with better delay of gratification skills. Finally, in two separate studies, FA values within the CC were found to be associated with tool use performance and intermanual transfer of tool use skills in chimpanzees (Phillips et al., 2013a,b). As has been reported in humans (Dunst et al., 2014; Yokota et al., 2022), we hypothesized that increasing performance on the PCTB task(s) would be positively associated with either streamline density or FA values for the CC.

## 2 Materials and methods

### 2.1 Subjects

A convenience sample of T1-weighted structural and diffusion tensor weighted images from 49 chimpanzees were downloaded from the National Chimpanzee Brain Resource.^1^ There were 32 females and 17 males in the sample and all of the apes had been previously housed at the Yerkes National Primate Research Center of Emory University. At the time they were scanned, the chimpanzees ranged between 9 and 54 years of age (Mean = 21.79 years, SD = 9.94). Chimpanzee hand preference classification was based on their hand use for a task assessing bimanual hand use, referred to as the tube task (Hopkins et al., 2013; Hopkins et al., 2011). A minimum of 50 hand use responses were recorded from each chimpanzee for this task. Hand preference classification was based on binomial z-scores performed on the frequency of left and right hand on the tube task. Chimpanzees were classified as left- (*z*-score = < −1.96), ambiguously- (*z*-score > −1.96 and <+1.96) or right-handed (*z*-score = > +1.96) from binomial z-scores performed on the frequencies in left and right hand use (Hopkins, 2013). Within this sample, there were 15 left-, 13 ambiguously- and 21 right-handed chimpanzees.

### 2.2 Image acquisition and post-image processing

All the scans reported in this publication were completed by the end of 2014 and have been used in previous dMRI studies (Bryant et al., 2020; Chauvel et al., 2023; Chen et al., 2013; Hecht et al., 2013; Hecht et al., 2017). Each individual was scanned on a 3 Tesla Trio MRI system (Siemens, Erlangen) using a bird-cage coil with a dedicated imaging protocol comprising anatomical (0.625 mm isotropic spatial resolution) and diffusion data (1.9 mm isotropic spatial resolution, *b* = 1000 s/mm^2^ single-shell acquisition with 60 diffusion directions, TE/TR = 86 ms/6 s, flip angle FA = 90°, read bandwidth RBW = 1563 Hz/pixel, matrix size 128 × 128, FOV = 243.2 mm). All procedures were carried out in accordance with protocols approved by the Emory University Institutional Animal Care and Use Committee.

Anatomical and diffusion MRI data were processed following previously described methods using a Python pipeline dedicated to the chimpanzee species developed with the CEA/NeuroSpin in-house C++ Ginkgo toolbox available at https://framagit.org/cpoupon/gkg (Chauvel et al., 2023; Herlin et al., 2023; Yebga Hot et al., 2022). This processing pipeline dedicated to the chimpanzee brain includes the correction for the various diffusion imaging artifacts present in dMRI individual datasets (Rician noise, eddy currents, susceptibility induced distortions). The computation of individual maps of local orientation distribution functions (ODF) were reconstructed from the diffusion-weighted volume using the analytical Q-ball model (Descoteaux et al., 2007). The computation of individual maps of local DTI models allows for quantification of FA, ADC, RD and MD maps and the inference of structural connectivity maps using the whole brain streamline regularized tractography algorithm from the former Q-ball maps (Perrin et al., 2005). Following the recommendation of Perrin et al. (2005), a whole-brain streamline regularized deterministic tractography algorithm was launched to each individual Q-ball ODF maps with the following parameters: 1 seed/voxel, forward step 0.4 mm, aperture angle 30°, lower GFA threshold = 0.15. Streamlines were generated within a propagation mask corresponding to the whole brain, established from the anatomical MRI using the Morphologist pipeline from BrainVisa (Fischer et al., 2012). This produced individual tractograms composed of several millions of fibers for each of the 49 chimpanzees.

### 2.3 Segmentation of white matter bundles and extraction of microstructural measures

**FIGURE 1:**
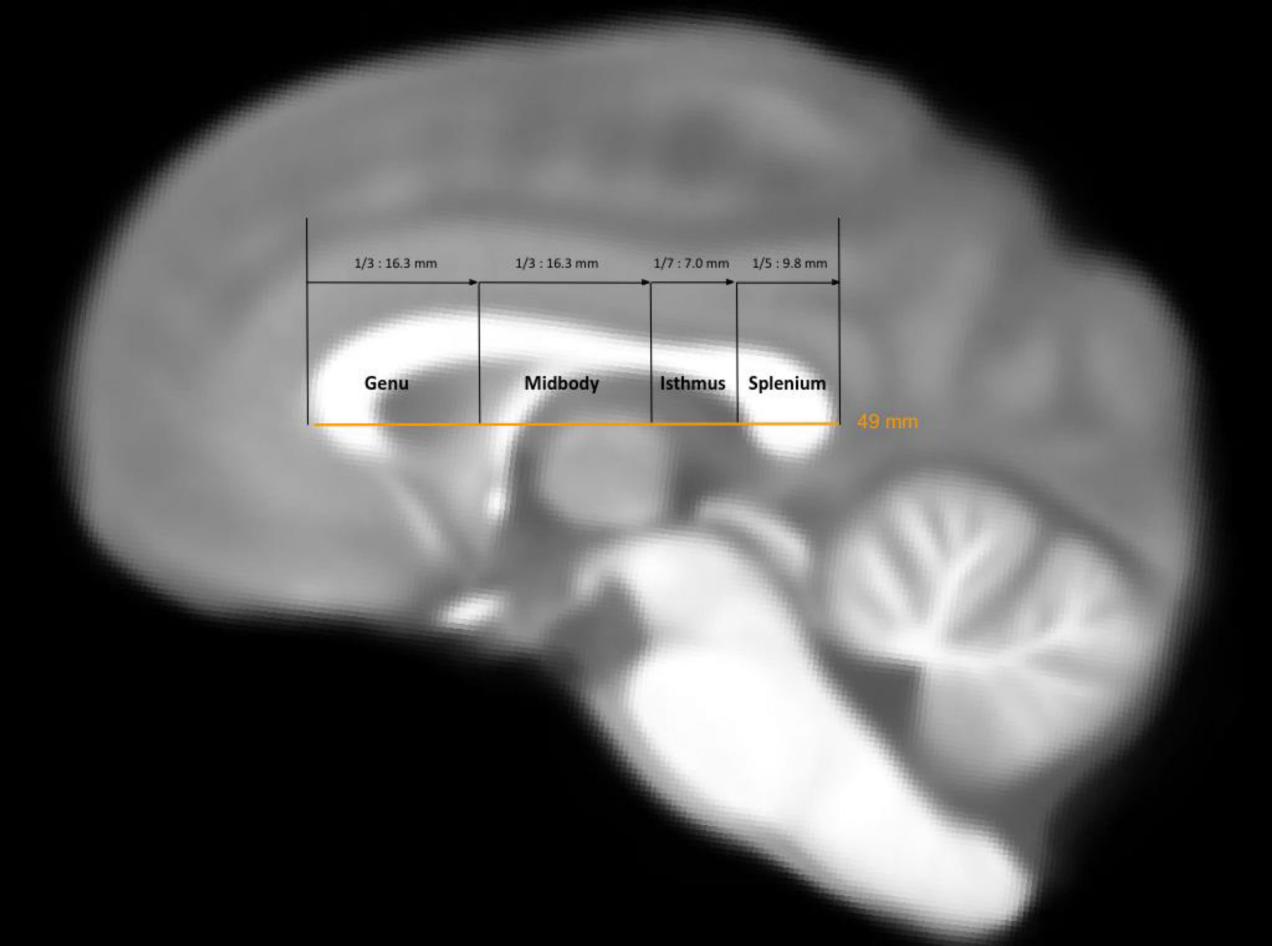
Midsagittal view of the chimpanzee brain showing the four CC regions of interest. Commissural regions are labeled using the Aboitiz labeling (Aboitiz et al., 1992) and are displayed on the Juna.Chimp template anatomical image (Vickery et al., 2020).

**FIGURE 2:**
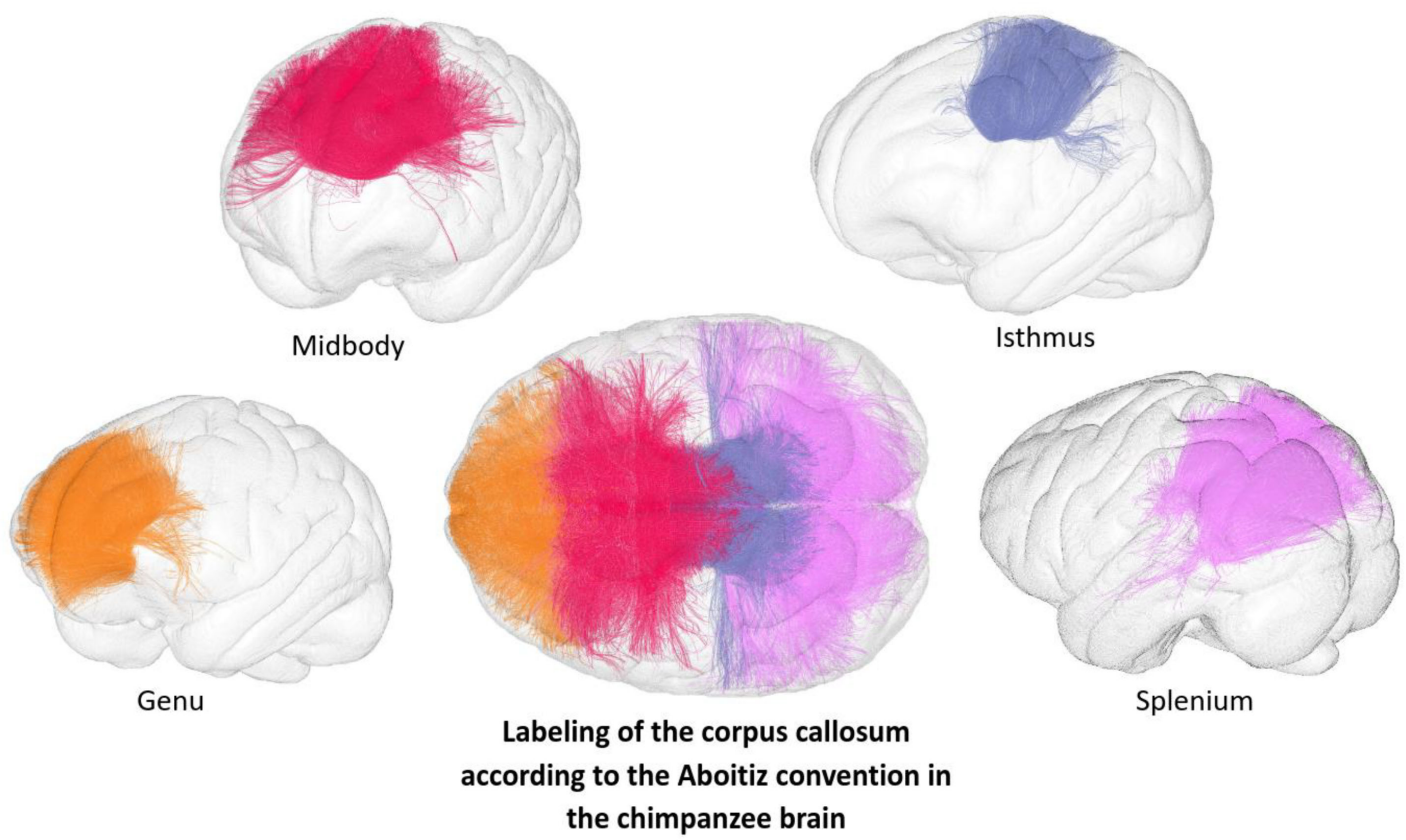
Commissural fiber regions using the Aboitiz labeling of the CC displayed on the Juna.Chimp template mesh. The central panel shows the full corpus callosum tracts labeled according to the Aboitiz convention, (Aboitiz et al., 1992) projected onto the cortical pial mesh of the Juna.Chimp chimpanzee brain template (Vickery et al. 2020). Surrounding panels display the subdivisions of the corpus callosum: genu (orange), midbody (magenta), isthmus (violet), and splenium (pink), each projected individually onto the cortical pial mesh.

Each individual anatomical MRI was matched to the Juna.Chimp chimpanzee template space developed by Vickery et al. (2020) using the Advanced Normalization Tools (ANTs) (Avants et al., 2009) to compute the corresponding non-linear diffeomorphic transformations between the template and individual radiological space. The resulting inverse transformation was then applied to the Ginkgo Chauvel chimpanzee brain deep white matter atlas (GCA) already built in the Juna.Chimp space to segment the 42 white matter tracts available in the Ginkgo “fiber labeling” tool. This GCA comprises corpus callosum subdivisions proposed by both Witelson (1985) and Aboitiz et al. (1992) (see Figures 1, 2). The four CC regions included in this analysis were the genu, anterior midbody, isthmus, and splenium. Fibers passing through the genu connect the frontal lobe while fibers passing through the midbody reach the posterior part of the frontal lobe, all of the parietal lobe and the insular lobes. Fibers passing through the isthmus reach the superior and posterior parietal lobe and those passing through the splenium reach the posterior part of the parietal lobe, the occipital lobe, and some parts of the temporal lobes (see Figure 2). From each subject and CC region, the number of streamlines reflecting the tract density was quantified. Additionally, mean values of axial diffusivity (AD), radial diffusivity (RD), fractional anisotropy (FA) and mean diffusivity (MD) were computed for each CC region. This was done using the “BundleMeasure” command from the Ginkgo toolbox, retrieving the DTI scalar values in each corresponding tract voxel. Further technical details about the white matter bundle segmentation process can be found in Chauvel et al. (2023).

### 2.4 Primate cognition test battery (PCTB)

The PCTB was originally developed by Herrmann et al. (2007) to assess comparative differences in physical and social cognition between human children, chimpanzees and orangutans. Here, we used PCTB data from a sample of 99 chimpanzees that were collected following the method described in Russell et al. (2011). In total, there are 13 tasks within the PCTB including 9 that assess physical cognition and 4 that assess social cognition. Physical cognition tasks include measures of spatial memory, object permanence, rotation, transposition, relative quantity discrimination, understanding of causality, tool properties and tool use. Social cognition measures include comprehension of pointing cues, initiation of pointing, the use of attention-getting behaviors and gaze following. Performance on each task was summed across trials and then converted to standardized *z*-scores. The *z*-scores were then averaged across the physical cognition and social cognition tasks to derive a unit weighted average performance score (herein, UWA). Higher UWA values reflected better cognitive performance across all tasks. PCTB data were available in all 49 chimpanzees on which DTI data were available. Collection of the PCTB data were obtained, on average, within 1.50 years (±SD = 1.68) of the dMRI (range −8 to +5 years).

### 2.5 Data analysis

To initially test for sex, handedness and age effects on the CC measures, analysis of covariance (ANCOVA) was performed with CC region as the repeated measure while sex and handedness grouping were the between group factors. Age at the time of the scan was the covariate. If age was found to have a significant effect on the CC measures, partial correlations were performed between these variables while controlling for sex and handedness to determine the direction of the association (Table 1). To guard against Type I error for all primary analyses, alpha was set to *p* < 0.025 after Bonferroni correction for multiple comparisons (adjusted *p* = 0.10/4). Note, because we had separate hypotheses regarding each DTI microstructural measure, separate Bonferroni estimates were applied to each set of analyses. Further, because we specifically hypothesized that age would have different directional associations with the CC measures, alpha was set to *p* = 0.10 instead of the typical two-tailed *p* = 0.05 (i.e., we adopted a one-tailed test). Notably, based on data from humans, we hypothesized that chimpanzees age would show significant negative associations with the streamline volume and FA values and positive associations with the AD, RD and MD values. Finally, to examine the relationship between cognitive performance and CC microstructure, we conducted partial correlation analyses between the UWA scores from the PCTB and the DTI-based measures (FA, AD, RD, MD) as well as streamline density. These analyses controlled for sex, handedness, and the difference in age between the cognitive testing session and the MRI scan. Correlation coefficients for each CC region and each microstructural measure are presented in Table 2. As with other analyses, a Bonferroni correction was applied across the four CC regions to account for multiple comparisons, with an adjusted alpha of *p* < 0.025. All statistical analyses were performed using SPSS.

**TABLE 1:** Partial linear correlation coefficients between age and corpus callosum (controlling for sex and handedness).

CC region	Streamline density	FA	AD	RD	MD
Genu	−0.330**	−0.145	−0.417**	−0.162	−0.275
Midbody	−0.329**	−0.126	−0.197	−0.192	−0.194
Isthmus	−0.245	−0.203	−0.132	−0.126	−0.128
Splenium	−0.185	−0.305**	−0.514**	−0.181	−0.359**

**Indicates significant at *p* < 0.025 (Bonferroni corrected). For all analyses, *N* = 49. FA, fractional anisotropy; AD, axial diffusivity; RD, radial diffusivity; MD, mean diffusivity.

**TABLE 2:** Partial correlation coefficients between intelligence and corpus callosum (controlling for sex, handedness and age).

CC region	Streamline density	FA	AD	RD	MD
Genu	+0.037	+0.346**	+0.219	−0.180	−0.078
Midbody	+0.059	+0.267*	+0.03	+0.029	+0.031
Isthmus	+0.019	+0.209	−0.146	−0.152	−0.150
Splenium	+0.144	+0.291*	+0.238	−0.094	+0.208

**Indicates *p* < 0.025 (corrected).

*p < 0.10. FA, fractional anisotropy; AD, axial diffusivity; RD, radial diffusivity; MD, mean diffusivity.

## 3 Results

### 3.1 Sex and handedness effects on CC microstructure and their association with age

**FIGURE 3:**
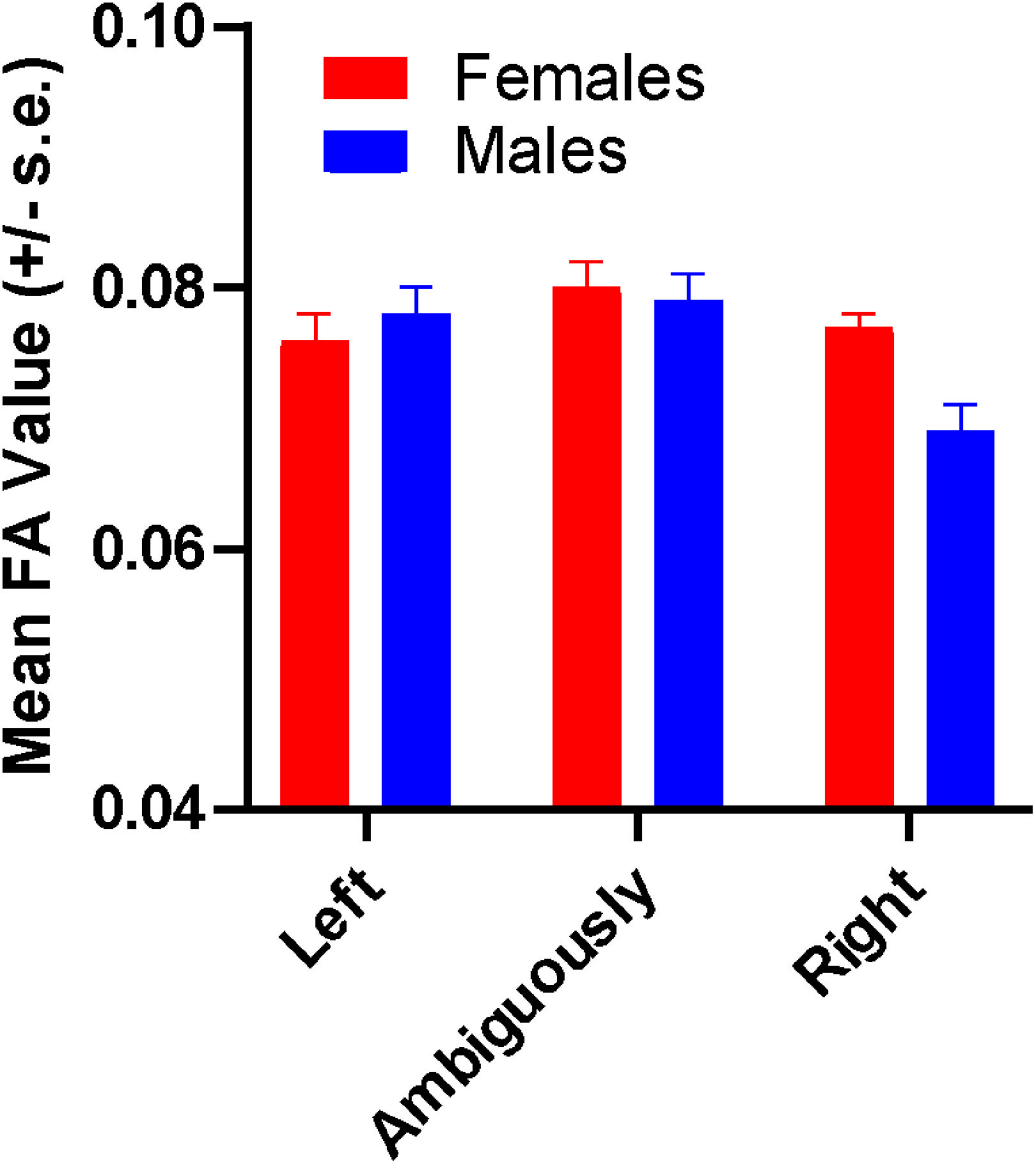
Mean FA values (±s.e) for left- ambiguously- and right-handed male and female chimpanzees.

**FIGURE 4:**
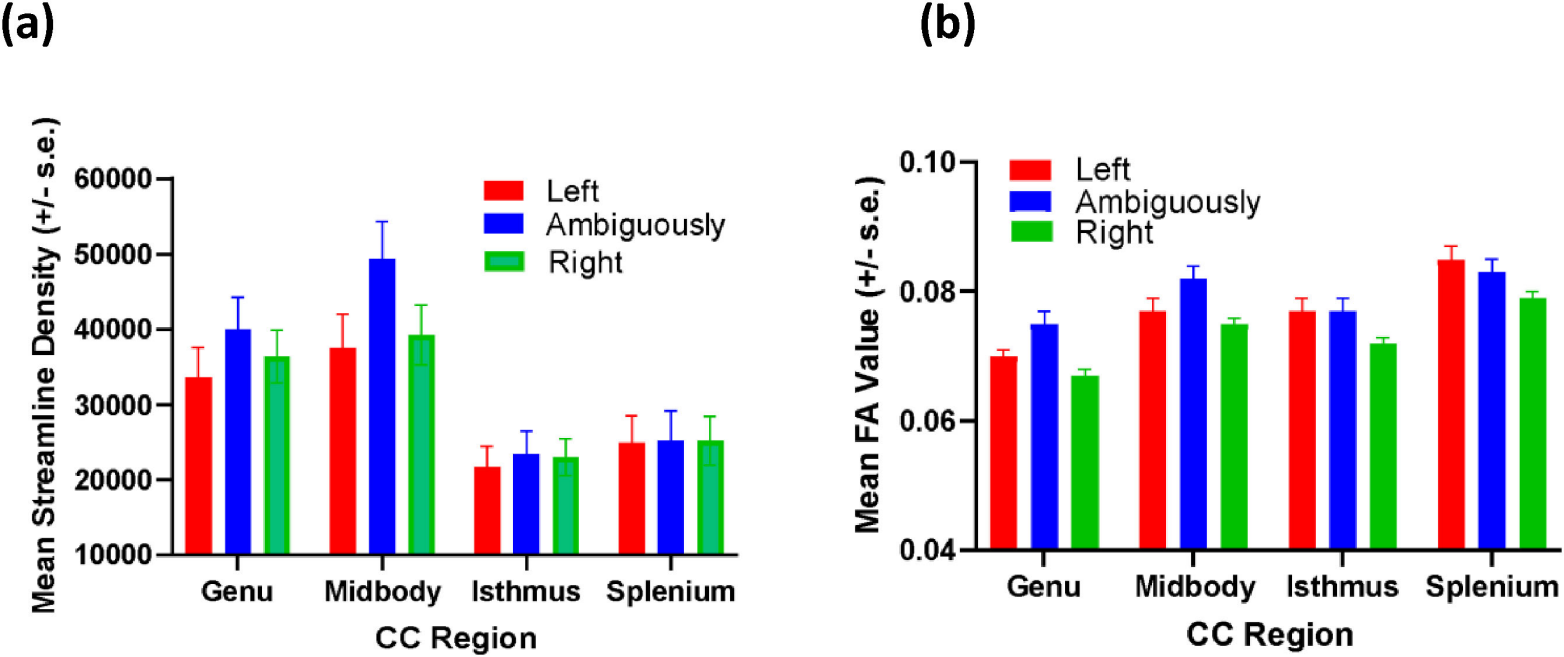
(a) Mean streamline density values (±s.e) for left- ambiguously- and right-handed chimpanzees for each CC region (b) Mean FA values (±s.e) for left- ambiguously- and right-handed chimpanzees for each CC region.

For FA, significant main effects were found handedness F(2, 42) = 5.563, *p* = 0.007, η^2^ = 0.210 and for the covariate age F(1, 42) = 3.994, *p* = 0.05, η^2^ = 0.087. Significant two-way interactions were found between handedness and CC region F(6, 126) = 2.859, *p* = 0.012, η^2^ = 0.120 as well as between sex and handedness F(2, 42) = 4.328, *p* = 0.020, η^2^ = 0.171. For the sex by handedness interaction (see Figure 3), *post hoc* analysis indicated that right-handed males had significantly lower FA values compared to ambiguously- and left-handed individuals. Among females, no significant differences in FA values were found for left-, ambiguously- and right-handed individuals. The mean FA values for left-, ambiguous- and right-handed chimpanzees as a function of CC region are shown in Figure 4a. *Post hoc* analysis indicated that ambiguously-handed chimpanzees had higher FA values in the genu and midbody compared to left- and right-handed apes. Because the covariate variable age was significant, partial correlation coefficients between age and the FA values for each CC region after controlling for sex and handedness were performed to determine the direction of the association (see Table 1). As shown in Table 1, age was significantly inversely associated with the splenium but none of the other CC regions.

For streamline density, a borderline significant main effect for the covariate age was found F(1, 42) = 3.702, *p* = 0.061, η^2^ = 0.081 as well as borderline significant interactions between handedness and CC region F(6, 126) = 2.004, *p* = 0.070, η^2^ = 0.087. As in the analysis of the FA values, partial correlation coefficient between age and CC region while controlling for handedness and sex were performed and these findings are shown in Table 1. Age was significantly negatively correlated with the genu and midbody but not the isthmus or splenium after controlling for handedness and sex. For the handedness and by CC region interaction, ambiguously-handed chimpanzees have higher streamline densities compared to left- and right-handed chimpanzees in the genu and midbody but not the isthmus or splenium (Figure 4b). The covariate age was not a significant main effect or interaction for any of the remaining measures; that said, we have nonetheless reported the associations between age and each CC region and measure while controlling for sex and handedness in Table 1.

### 3.2 Partial correlation between cognition and CC microstructure

We next considered the association between the UWA scores and the CC streamline density, FA, AD, MD and RD measures while statistically controlling for sex, handedness and the difference in age between the scan collection and cognitive testing age. These results are shown in Table 2. The only correlation that survived correction to alpha was a significant positive association between UWA scores and genu FA values (*p* < 0.025, corrected).

## 4 Discussion

The results of this study are straightforward. With respect to streamline density, as hypothesized, older chimpanzees had fewer interhemispheric fibers connecting prefrontal and premotor regions of the chimpanzee brain, notably the genu and anterior midbody. For FA, increasing age was inversely associated with the splenium but no other regions. Further, for axial diffusivity, increasing age was associated with lower values in the genu and splenium, though neither the main effect for age nor the interaction between age and CC region were significant. Significant handedness effects were also found for the streamline densities and FA values that were specific to certain CC regions. Ambiguously-handed chimpanzees had higher FA values and increased streamline densities for the genu and midbody compared to left- and right-handed apes. These findings are consistent with theories postulating that more lateralized individuals (and species) have decreasing levels of interhemispheric connectivity (Aboitiz et al., 2003; Aboitiz et al., 1992; Caminiti et al., 2013; Hopkins and Cantalupo, 2008; Luders et al., 2010; Oliveras et al., 2001; Rilling and Insel, 1999; Ringo et al., 1994).

Although FA is widely used in studies of white matter microstructure, it lacks specificity to underlying biological processes such as axon density, myelination, or fiber coherence (Figley et al., 2022). Therefore, we also examined streamline density, axial diffusivity (AD), radial diffusivity (RD), and mean diffusivity (MD) to provide a broader perspective on CC microstructure. While only a subset of these measures showed statistically significant effects, the overall pattern contributes to a more comprehensive understanding of aging and individual variation in chimpanzee white matter. Overall, the results with respect to aging show both consistent and inconsistent patterns with findings previously reported in human subjects. On the one hand, as has been reported in humans, streamline density was inversely associated with age in chimpanzees, particularly in the anterior and central regions of the CC. For the FA, RD and AD measures, the results from chimpanzees differ from the general pattern of results reported in humans. Notably, in humans, negative associations between FA and age are typically stronger in the anterior compared to posterior regions of the CC. By contrast, in the chimpanzees the opposite pattern was found with the only significant association being in the splenium (at least for FA). This suggests that the integrity of interhemispheric connectivity in frontal cortical brain regions is stronger in chimpanzees compared to humans. Interestingly, recent evidence in humans has questioned the link between handedness and corpus callosum morphology. Westerhausen et al. (2025) reported no significant associations between hand preference and CC morphology or hemispheric language dominance, suggesting that the structural correlates of lateralization may be more complex than previously assumed. Our findings in chimpanzees, by contrast, indicate that handedness–particularly ambiguous-handedness–is associated with differences in FA and streamline density in the anterior CC. Further, in humans, both radial and axial diffusivity are positively associated with increasing age whereas in the chimpanzee, the opposite pattern was found with age inversely correlating with both measures, particularly in the anterior CC regions. Because RD and AD have been proposed to reflect myelination, axonal damage and axonal density, these findings suggest that elderly chimpanzees do not experience reductions in axon density, damage or myelination loss. Thus, like previous findings on CC surface area and thickness (Westerhausen et al., 2021), there is a marked absence in loss of CC microstructural organization in aged chimpanzees, at least within this sample (Bennett and Madden, 2014; Coelho et al., 2021; Li et al., 2020). These findings are also somewhat consistent with reports of relatively small loss in neuron counts in aged chimpanzees (Edler et al., 2020).

While chimpanzees do show age-related decline in cognition (as measured by the PCTB), the slope in change overtime is relatively modest compared to humans (Hopkins et al., 2021; Lacreuse et al., 2014). The findings reported here may explain, in part, the lack of substantial loss in cognitive functions in chimpanzees with increasing age compared to humans. In support of this argument are the significant associations found between the UWA scores and the FA values after controlling for age, handedness and sex (see Table 2). Chimpanzees with higher UWA scores had higher FA in the genu, the CC region largely connecting prefrontal and premotor regions. Thus, more intelligent apes had corpus callosi that were higher in integrity despite having fewer fibers. These collective findings suggest that, though elderly chimpanzees show loss in gray matter volume and thickness with age, they may avoid significant loss in cognitive functions by retaining increased connectivity between regions involved in different functions. This interpretation is somewhat consistent with the disconnection theory of Alzheimer’s disease, at least as it pertains to interhemispheric connectivity (Bennett and Madden, 2014).

## 5 Limitations

There are at least four limitations to this study. First, the sample size was relatively small, particularly among older male subjects. The inclusion of a larger sample of males would be useful as a means of better assessing the impact of sex on CC microstructure. Second, as is the case with all cross-sectional studies, the current studies only reflect age group differences not age-related changes in CC microstructure. Like in studies with humans, cross-sectional studies in non-human primates are similarly subject to cohort effects. Longitudinal changes in CC microstructure would remove the potential for cohort effects but, unfortunately, *in vivo* MRI scanning of chimpanzees is no longer permitted in light of their endangered species status. Thus, longitudinal studies on changes in CC microstructure will require the use of other primate model species. Fourth, while the current findings are discussed in comparison to humans, it is important to acknowledge that environmental and lifestyle differences between species such as diet, exposure to disease, physical activity, and medical care could influence the trajectory of brain and cognitive aging and are difficult to estimate. Lastly, the effect sizes were relatively small in all reported associations between age and CC microstructure. This suggests that factors other than age, sex and handedness likely contribute to variation in CC microstructure.

## 6 Conclusion

In summary, the findings reported here provide further data on the neurobiology of aging in primates and is one of the first studies to focus on age-related differences in white matter connectivity in male and female chimpanzees. Further studies should focus on expanding the sample sizes with the goal of equal representation of males and females within the study. Further, DTI can be used with postmortem brains and, in these circumstances, acquired at much higher voxel resolution. There are more than 300 postmortem brains within the National Chimpanzee Brain Resource^1^ and these available specimens could be used in future DTI studies designed to further examine the comparative biology of aging in human and non-human primates with increased rigor and statistical power.

## Data Availability

The original contributions presented in this study are included in this article/supplementary material, further inquiries can be directed to the corresponding author.

## Footnotes

1
www.chimpanzeebrain.org
